# Emergence and potential transmission route of avian influenza A (H5N1) virus in domestic cats in Poland, June 2023

**DOI:** 10.2807/1560-7917.ES.2023.28.31.2300390

**Published:** 2023-08-03

**Authors:** Lukasz Rabalski, Aleksandra Milewska, Anne Pohlmann, Karolina Gackowska, Tomasz Lepionka, Klaudiusz Szczepaniak, Agnieszka Swiatalska, Izabela Sieminska, Zbigniew Arent, Martin Beer, Marion Koopmans, Maciej Grzybek, Krzysztof Pyrc

**Affiliations:** 1 Intercollegiate Faculty of Biotechnology of University of Gdansk and Medical University of Gdansk, Gdansk, Poland; 2 Biological Threats Identification and Countermeasure Centre, Military Institute of Hygiene and Epidemiology, Pulawy, Poland; 3 Małopolska Centre of Biotechnology, Jagiellonian University, Kraków, Poland; 4 Institute of Diagnostic Virology, Friedrich-Loeffler-Institut, Greifswald-Insel Riems, Germany; 5 Faculty of Veterinary Medicine, University of Life Sciences in Lublin, Lublin, Poland; 6 Private Veterinary Clinic, Warzento, Poland; 7 University of Agriculture in Krakow, Krakow, Poland; 8 Department of Viroscience, Erasmus MC University Medical Center, Rotterdam, the Netherlands; 9 Institute of Maritime and Tropical Medicine, Medical University of Gdańsk, Gdynia, Poland

**Keywords:** bird flu, influenza, biomonitoring, domestic cats, companion animals, meat contamination, feed safety

## Abstract

In June 2023, a fatal disease outbreak in cats occurred in Poland. Most cases tested in Poland (29 of 47) were positive for highly pathogenic avian influenza (HPAI) A (H5N1) virus. Genetic analyses revealed clade 2.3.4.4b with point mutations indicative of initial mammalian hosts adaptations. Cat viral sequences were highly similar (n = 21), suggesting a potential common infection source. To investigate possible infection routes, our group tested food samples from affected households. HPAI H5N1 virus was detected in one poultry meat sample.

Since their emergence in poultry in Guangdong, China, highly pathogenic avian influenza A (HPAI) subtype H5N1 viruses have undergone a series of complex reassortments, leading to the development of multiple descendant lineages. Notably, starting December 2022 the clade 2.3.4.4.b has caused a widespread and unprecedented outbreak on a global scale among wild birds [1].

While HPAI H5N1 viruses are recognised as posing a risk for zoonotic disease and have been associated with severe illness, only 12 human cases attributed to the 2.3.4.4.b clade have been reported so far to the World Health Organization, including four cases of severe illness [2]. Following increasing numbers of cases observed among wild and farmed mammals, there has been a concern about spillover infections from wild birds to mammals. The widespread opportunity for exposure of mammals created by the worldwide dissemination of the HPAI H5N1 2.3.4.4.b viruses among wild birds also raises the issue of potential adaptation of HPAI H5N1 viruses, which could lead to their facilitated transmission among mammals [3-6].

Here, our group presents evidence of infections in domestic cats in Poland, which constitute a potential risk to both pet owners and veterinarians. As the sudden reporting of multiple cases across the country (28 domestic cats and one captive caracal [2]) is highly unusual, we conducted tests on cat food as a potential source of infection and successfully detected infectious HPAI H5N1 virus.

## Detection of genetic material of influenza A (H5N1) virus in cats

Infections with HPAI H5N1 virus have the potential to cause acute and severe disease in cats [7], and while mild and asymptomatic infections have been observed, the virus was also reported to cause encephalitis and ganglioneuritis in domestic cats and wild feline species [8]. Moreover, multifocal haemorrhages and necrosis across different organs, along with bronchointerstitial pneumonia have been observed to contribute to the mortality observed in HPAI H5-affected animals [8]. 

On 19 June 2023, we were first alerted by cat owners and veterinarians in Poland about a fatal disease of unknown origin in cats occurring in the country. Because HPAI H5N1 virus was suspected as a possible cause for the illness, swabs from four cats showing symptoms were collected by veterinary clinicians during clinical procedures and we tested for HPAI H5N1 virus. The analysis revealed the presence of HPAI H5 virus in samples tested from all four animals (Table).

**Table:** Results of influenza-specific RT-qPCR on cat-derived samples^a^, Poland, June 2023 (n = 4 cats)

Cat	Cat origin and identifier	Specimen	RT-qPCR Cq	
			M	H5
1^b^	Kra1	Nasal swab^c^	29.77	32.93
		Nasopharyngeal swab	36.63	>35
		Serum	>42	>35
2	Gda1	Nasal swab^c^	19.34	27.51
3^b^	Gda2	Nasal swab	40.55	>35
4	Gda3	Nasal swab	33.37	>35

Cq: quantification cycle; Gda: Gdańsk; Kra: Kraków; M: matrix gene PCR target; H5: haemagglutinin of the subtype 5 PCR target; RT-qPCR: reverse-transcription-quantitative PCR; WHO: World Health Organization.

^a^ RT-qPCR on M gene (WHO information for the molecular detection of influenza viruses [16]).

^b^ Samples from animals that recovered from their illness.

^c^ Samples that were sequenced with partial or full genomes.

Further to this, more tests were performed in the country and most animals tested (29 of 47), including 28 domestic cats and one captive caracal, were found positive for HPAI H5N1 [2].

## Geographical distribution estimated from cat owner reports

**Figure 1:**
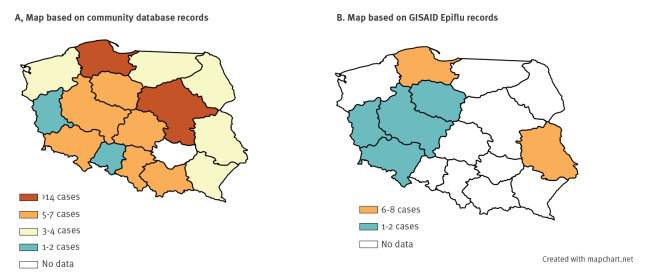
Geographical distribution of (A) ill cats or (B) cat cases confirmed with HPAI H5N1 virus infection, across Polish voivodships, June 2023

Following veterinarians’ notifications of H5N1 HPAI virus infection in cats, on 23 June 2023 a group of cat owners created a public database, enabling people with cats suffering from serious disease to deposit their cats’ records [9,10]. The database is not curated and is not an official source, but we explored it as a citizen science dataset. The database listed 89 entries as accessed on 12 July, and revealed a nationwide distribution of cases across Poland, with no discernible pattern (Figure 1). The outbreak also affected both indoor and outdoor cats.

HPAI: highly pathogenic avian influenza.

This map of Poland illustrates the spatial distribution of reported cases in different voivodships reported in June 2023.

Panel A presents the suspected case distribution as per the community-based database records, whereas Panel B depicts the distribution of cases as confirmed by the GISAID Epiflu records.

The number of records began to rise after the first week of June, peaking in the third week of this month and then decreasing towards the month's end. The interval from onset of clinical signs to death reported by the cat owners ranged from 0 to 7 days. As illustrated in Supplementary Figures 2−5, the most frequently reported symptoms included dyspnoea, cognitive impairment, seizures, limb rigidity, non-reactive pupils, anisocoria, decreased oxygen saturation, fever, loss of appetite, and hyperglycaemia. While these records are not validated, they fit well with the symptoms and disease course of the cats we diagnosed, as detailed in the Supplementary Table 3.

## Analysis of viral genetic material retrieved from cats

Samples from cats testing positive for HPAI H5N1 virus were submitted for Oxford Nanopore Technologies' (ONT) MinION whole genome sequencing. Whole virus segments were reverse transcribed and amplified and sequenced using the ONT Ligation sequencing influenza whole genome protocol with some changes [11] as listed in the Supplementary Materials and Methods. The viral sequences obtained from two of the four cats investigated by us were deposited in GISAID EpiFlu database under accession numbers: EPI_ISL_17949824 (A/cat/Poland/Gda1/2023; whole genome sequence), EPI_ISL_ 17989196 (A/cat/Poland/Kra1/2023; partial genome but with full haemagglutinin (HA) and neuraminidase (NA) gene sequences). In addition, publicly available genomes from databases, which are described in Supplementary Table 1 were used for the phylogenetic analysis shown in Supplementary Figure 1.

Our analysis suggests that all of the HPAI H5N1 viruses we (n = 2) and the Polish National Veterinary Research Institute (n = 19) isolated from cats in Poland belong to clade 2.3.4.4b and cluster with virus strains from birds sampled in Central Europe from late 2022 onwards. Viral sequences in this cluster show a distinct segment composition with HA and NA segments similar to sequences from H5N1 HPAI viruses from the current panzootic H5 clade 2.3.4.4b and five other segments (PB2, PB1, PA subunits, matrix protein (MP) and non-structural (NS) protein) from viruses circulating since late 2021 [5]. They include a distinct nucleoprotein (NP) segment found from late 2022 onwards (Supplementary Figure 1). 

**Figure 2:**
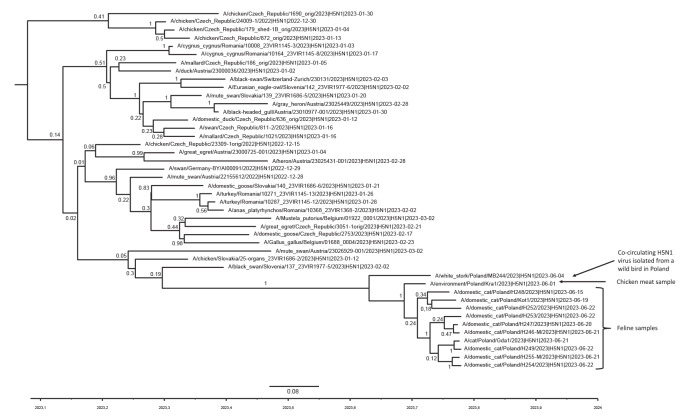
Time-scaled maximum clade credibility (MCC) phylogeny of concatenated coding sequences of HPAI H5N1 viruses of clade 2.3.4.4b collected in Europe in 2023

A time-scaled phylogeny (Figure 2) confirms the appearance and spread of this genotype in Central Europe in 2023. This assessment also reveals a gap in the available molecular sequence data for the period immediately preceding the abnormal deaths in cats, which limits the ability to analyse and clarify relationships. Moreover, bias due to incomplete sequence data reporting by countries cannot be ruled out. The phylogenetic tree includes data from poultry outbreaks; the corresponding genotype was found in several holdings in different countries outside Poland (Figure 2).

HPAI: highly pathogenic avian influenza.

Posterior probabilities are given at the branch level. In the tree, the genetic sequences retrieved in the current study are the following: A/cat/Poland/Gda1/2023 (EPI_ISL_ 17989196), and A/environment/Poland/Kra1/2023 (EPI_ISL_17959737). For readability, not all cat sequences, which were identical or near-identical are displayed.

The analysed HPAI H5N1 virus genomes from cats are almost identical as shown in Supplementary Table 2, suggesting a monophyletic origin and a potential common source of infection. They all carry mutations associated with mammalian adaptation in the polymerase gene (both PB2-E627K and PB2-K526R), allowing for replication at lower temperatures [12]. An assessment of both the HA and NA genes found no other changes of concern in the sequenced viruses.

The identification of avian influenza viruses with mammalian adaptation markers in several infected cats could indicate rapid adaptation of viruses of this clade in each of the infected animals. However, the simultaneous occurrence of two mutations makes this a less likely scenario. It appears that the PB2-E627K mutation was found in a HPAI H5N1 virus sequence from a wild white stork in Poland (A/white_stork/Poland/MB244/2023), which in the tree clusters with the viruses derived from cats. One could thus hypothesise that the virus may have already had this mutation in an avian host. Notably, the PB2-E627K change was suggested to be unfavourable for the virus replication in avian hosts [13]. The circulation of viruses with mammalian adaptation markers among wild birds is remarkable and could indicate spillback following circulation in a mammalian host where an initial adaptation took place. More in depth studies are needed to elucidate the chain of events.

## Investigation of potential transmission route

Next, our research focused on discerning the possible transmission pathway of the virus from birds to felines. From an epidemiological perspective, several features merit attention: first, the remarkable similarity among the sequences of clinical samples could be indicative of a monophyletic origin; second, the stochastic distribution of the virus within Poland and the absence of similar cases noted in neighbouring countries imply a specific geographical determinant; third, the citizen science data collection suggested that both indoor and outdoor cats have contracted the infection, narrowing the scope of potential transmission routes. These observations, taken together, could suggest a common exposure, potentially through cat food, as has previously been documented for HPAI H5N1 virus [14]. To investigate this further, we reached out to owners of cats that recently experienced severe disease consistent with HPAI H5N1 virus infection. Specifically, we sought samples of the food that these animals consumed prior to the emergence of symptoms.

We received five frozen meat samples and detected high levels of viral RNA in one of them, which was chicken meat that had been purchased fresh for human consumption on 9 June 2023 (Supplementary Materials and Methods). The quantification cycle (Cq) values were as low as 20 for the matrix (M) gene and 25 for the HA gene. Whole genome sequencing of this sample (A/environment/Poland/Kra1/2023) confirmed its high identity with maximum two differences at nucleotide level per segment to the viruses isolated from the cats (GISAID: EPI_ISL_17959737; Figure 2, Supplementary Figure 1, and Supplementary Table 2). The viral sequence obtained from the chicken had the PB2-E627K and PB2-K526R mutations.

## Cytopathic and replication ability of the virus in cell cultures

The remaining diluted meat juice was then inoculated onto Madin−Darby canine kidney (MDCK) cells, a standard procedure for influenza A virus isolation (Supplementary Materials and Methods). After 72-hour culture, a distinct cytopathic effect was noted. The isolate's identity was verified through reverse-transcription quantitative PCR (RT-qPCR). Subsequently, the first passage (p1) virus stock was titrated on MDCK, Crandell−Rees feline kidney (CRFK), *Felis catus* whole fetus (FCWF) and Vero cells respectively. A cytopathic effect was observed on all cell lines 24 hours post-inoculation as illustrated in Supplementary Figure 6. Using the Reed and Muench method, the virus titre expressed as 50% tissue culture infectious dose per mL (TCID_50_/mL) was determined to be 8.7 × 10^6^ for MDCK cells, 9.3 × 10^3^ for CRFK cells, 1.4 × 10^4^ for FCWC cells, and 1.3 × 10^7^ TCID_50_/mL for Vero cells, the last of which was collected 48 hours post-infection.

**Figure 3:**
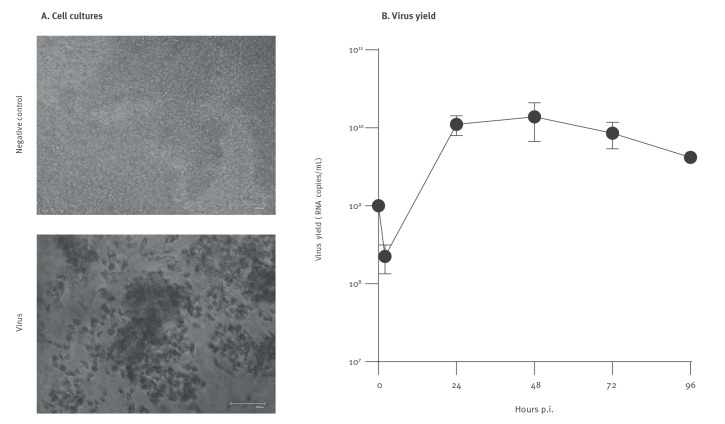
Influenza A (H5N1) clade 2.3.4.4.b infection in human airway epithelium, Poland, June 2023

Finally, we evaluated the replication competence in fully differentiated human airway epithelial cultures (Epithelix SAS, Archamps, France), as variable pathogenicity in human tissue was previously shown for different H5N1 2.3.4.4.b isolates [15]. Briefly, cultures were inoculated with the p1 virus stock and monitored for 4 days at 37°C. By day 3 post-infection, a pronounced cytopathic effect and tissue damage was evident in virus-inoculated cultures (Figure 3A), as confirmed by RT-qPCR (Figure 3B).

HPAI: highly pathogenic avian influenza; p.i.: post inoculation.

A. Cells were inoculated with passage 1 HPAI H5N1 virus isolated from the chicken meat and cultured for 96 hours. Cytopathic effect was observed in virus-infected cultures. RT-qPCR was used to determine the virus yield each day p.i.

B. Samples were analysed in four biological replicates.

## Discussion

We report the detection and genetic analysis of HPAI H5N1 viruses in ill cats in Poland, within the context of a fatal disease outbreak that occurred in this country in June 2023. Based on an unofficial database created by cat owners, the distribution of cat cases suspected of being related to the outbreak appeared to be nationwide. Phylogenetic analyses suggest a monophyletic origin of the viruses affecting the cats, which were characterised as belonging to clade 2.3.4.4b with two mutations (E627K and K526R) in PB2, considered to be mammalian adaptation markers. Based on available data, the cat-derived viral sequences appeared closely related to an HPAI H5N1 virus sequence derived from a stork, which was also clade 2.3.4.4b. This sequence nevertheless only bore the K526R mutation.

Reaching out to owners whose cats had experienced an illness compatible with HPAI H5N1 infection, some samples from food that these animals had reportedly consumed prior to the emergence of symptoms were obtained and tested. A virus with high degree of similarity to the cat viruses and bearing the E627K and K526R was found in chicken meat from the same household as an ill cat, suggesting, but not confirming, a possible route of transmission. An HPAI H5N1 virus isolate from the chicken meat juice demonstrated the ability to infect both canine and feline cells, and efficiently infected and damaged human airway epithelia in cell culture.

This study has some limitations. The virus found in a chicken meat sample originated from a private refrigerator of one of the cat owners; its origin cannot be confirmed, and it is essential to consider that it might have been contaminated post-butchery, during transport, or at the household. The meat samples under investigation were sourced from households where severe illness was reported in cats. However, these cases were not conclusively identified as being infected by HPAI H5N1 virus; it is also important to note that the meat samples analysed were not necessarily the sole variety of meat these cats consumed. The public database mentioned in this report relied upon is a community initiative by cat owners that has not undergone any form of curation. Caution should therefore be exercised when interpreting the data. Nevertheless, in the early stages of an outbreak, such an initiative can swiftly provide critical information and serve as a valuable resource.

### Conclusion

Considering the presented data, we recommend that the presence of the virus should be assessed in both wild and farmed settings and other locations where potential virus adaptation may occur. Taking into account non-food-related routes of transmission, environmental testing may be recommended, as well as testing of wild birds with no apparent pathology. Further epidemiological analysis might reveal possible links between the cases and, subsequently, shed more light on the source of infection.

## Supplementary Data

521000 SupplementaryMaterialsAndMethods

## Supplementary Data

933000 SupplementaryFigures

## Supplementary Data

38000 SupplementaryTables

## References

[1] Centers for Disease Control and Prevention (CDC). Technical Report: Highly Pathogenic Avian Influenza A(H5N1) Viruses. Atlanta: CDC; Updated 7 Jul 2023. [Accessed 2 Aug 2023]. Available from: https://www.cdc.gov/flu/avianflu/spotlights/2022-2023/h5n1-technical-report_june.htm#human-cases

[2] World Health Organization (WHO). Influenza A(H5N1) in cats – Poland. Geneva: WHO. 16 July 2023. Available from: https://www.who.int/emergencies/disease-outbreak-news/item/2023-DON476

[3] Alkie TN Cox S Embury-Hyatt C Stevens B Pople N Pybus MJ Characterization of neurotropic HPAI H5N1 viruses with novel genome constellations and mammalian adaptive mutations in free-living mesocarnivores in Canada. Emerg Microbes Infect. 2023;12(1):2186608. 10.1080/22221751.2023.2186608 36880345 PMC10026807

[4] Bordes L Vreman S Heutink R Roose M Venema S Pritz-Verschuren SBE Highly Pathogenic Avian Influenza H5N1 Virus Infections in Wild Red Foxes (Vulpes vulpes) Show Neurotropism and Adaptive Virus Mutations. Microbiol Spectr. 2023;11(1):11. 10.1128/spectrum.02867-22 PMC9927208 36688676

[5] Vreman S Kik M Germeraad E Heutink R Harders F Spierenburg M Zoonotic Mutation of Highly Pathogenic Avian Influenza H5N1 Virus Identified in the Brain of Multiple Wild Carnivore Species. Pathogens. 2023;12(2):168. 10.3390/pathogens12020168 36839440 PMC9961074

[6] Siegers JY Ferreri L Eggink D Kroeze EJBV te Velthuis AJW van de Bildt M Evolution of highly pathogenic H5N1 influenza A virus in the central nervous system of ferrets. PLoS Pathog. 2023;19(3):e1011214. 10.1371/journal.ppat.1011214 36897923 PMC10032531

[7] Kuiken T Rimmelzwaan G Van Riel D Van Amerongen G Baars M Fouchier R Avian H5N1 influenza in cats. Science. 2004;306(5694):241. 10.1126/science.1102287 15345779

[8] Frymus T Belák S Egberink H Hofmann-Lehmann R Marsilio F Addie DD Influenza Virus Infections in Cats. Viruses. 2021;13(8):1435. 10.3390/v13081435 34452300 PMC8402716

[9] Choroba kotów (Odpowiedzi) - Arkusze Google. [Feline disease (answers) - Google Sheets]. Polish. [Accessed 12 Jul 2023]. Available from: https://docs.google.com/spreadsheets/d/1mxrfKbes4JoEDyxSisYFPindgd_N7Mer9e7KM7biIxo/edit?fbclid=IwAR1k30LBVVN3s-5LOwI5dOWKUNaNwcZsaR0DUVgO5DZRJaMwFBGXuyo_nvQ#gid=1893899732

[10] Miau.PL • Zobacz wątek - Tajemnicza śmiertelna choroba kotów. [See the topic - Mysterious deadly feline disease]. Polish. [Accessed 12 Jul 2023]. Available from: https://forum.miau.pl/viewtopic.php?p=12742263

[11] Oxford Nanopore Technologies (ONT). ONT Ligation sequencing influenza whole genome protocol. Oxford: ONT. [Accessed 13 Jul 2023]. Available from: https://community.nanoporetech.com/docs/prepare/library_prep_protocols/ligation-sequencing-influenza-whole-genome/v/inf_9166_v109_revb_24aug2022

[12] Song W Wang P Mok BWY Lau SY Huang X Wu WL The K526R substitution in viral protein PB2 enhances the effects of E627K on influenza virus replication. Nat Commun. 2014;5(1):5509. 10.1038/ncomms6509 25409547 PMC4263149

[13] Bogs J Kalthoff D Veits J Pavlova S Schwemmle M Mänz B Reversion of PB2-627E to -627K during replication of an H5N1 Clade 2.2 virus in mammalian hosts depends on the origin of the nucleoprotein. J Virol. 2011;85(20):10691-8. 10.1128/JVI.00786-11 21849466 PMC3187502

[14] Harder TC Teuffert J Starick E Gethmann J Grund C Fereidouni S Highly pathogenic avian influenza virus (H5N1) in frozen duck carcasses, Germany, 2007. Emerg Infect Dis. 2009;15(2):272-9. 10.3201/eid1502.080949 19193272 PMC2657628

[15] Kandeil A Patton C Jones JC Jeevan T Harrington WN Trifkovic S Rapid evolution of A(H5N1) influenza viruses after intercontinental spread to North America. Nat Commun. 2023;14(1):3082. 10.1038/s41467-023-38415-7 37248261 PMC10227026

[16] World Health Organization (WHO). WHO information for the molecular detection of influenza viruses. Geneva: WHO; 2021. [Accessed 12 Jul 2023]. Available from: http://www.who.int/influenza/gisrs_laboratory/collaborating_centres/list/en/index.html

