# Supplementary material for: Emergence and potential transmission route of avian influenza A (H5N1) virus in domestic cats in Poland, June 2023
Source: Euro Surveill. 2023 Aug 3;28(31):2300390. doi: 10.2807/1560-7917.ES.2023.28.31.2300390 (PMC10401914; doi:10.2807/1560-7917.ES.2023.28.31.2300390)
Supplement: SupplementaryMaterialsAndMethods [file 2300390_SuplementaryMaterialsAndMethods.pdf]

This supplementary material is hosted by Eurosurveillance as supporting information alongside the article "Emergence and potential transmission route of avian influenza A (H5N1) in domestic cats in Poland, June 2023", on behalf of the authors, who remain responsible for the accuracy and appropriateness of the content. The same standards for ethics, copyright, attributions and permissions as for the article apply. Supplements are not edited by Eurosurveillance and the journal is not responsible for the maintenance of any links or email addresses provided therein.

Supplementary Materials and Methods.

RT-qPCR

Swabs collected from cats were mixed with the RLT lysis buffer and total nucleic acids were isolated, as advised by the manufacturer (RNeasy Mini Kit; QIAGEN). Resulting samples were tested immediately or stored at -80°C for further analysis. The RNA was then tested in a RT-qPCR reaction using a TaqPath 1-Step Master Mix (ThermoFisher Scientific) with primers and probes for the H5 gene (Influenza Virus Real-Time RT-PCR Assay; BEI Resources, cat number NR-15592) and matrix (M) gene (WHO information for the molecular detection of influenza viruses[9]) in white, 8-well, quantitative PCR strips with optical clear caps (Applied Biosystems). The signal was evaluated using a Light Cycler 480 apparatus (Roche) (2 min at 25°C, 10 min at 53°C, 2 min at 95°C, followed by 45 cycles 3 s at 95°C and 30 s at 60°C).

meat samples, which we defrosted and incubated in saline for 15 minutes at room temperature. Part of the resultant supernatant was mixed with R9F buffer at a 1:4 v/v ratio, while the rest was stored at -80°C. We isolated total nucleic acids (Viral RNA/DNA; A&A Biotechnology) and conducted RT-qPCR as described above.

RNA sequencing

RNA was sequenced using Oxford Nanopore Technologies' (ONT) MinION. Whole influenza virus segments were reverse transcribed and amplified employing ONT Ligation sequencing influenza whole genome protocol with changes[1]. In brief, isolated total RNA was used for single-step RT-PCR reaction with universal primers by Bin Zhoue et al. and SuperScript III One-Step RT-PCR System with Platinum Taq DNA Polymerase (ThermoFisher)[2] Further, amplified DNA fragments were visually confirmed on agarose gel electrophoresis. Even samples that do not show clear bands, but only smear were used for the next step, which was the library preparation using the Rapid Barcoding Kit (SQK-RBK004) instead of Ligation Sequencing Kit (SQK-LSK109) and Native Barcoding Expansion 96 (EXP-NBD196) as it is stated in manufacturer's protocol. The pooled barcoded libraries were cleaned up using AMPure XP beads (Beckman Coulter Diagnostics, CA, USA) in a 1:1 ratio and eluted in 10 µL elution buffer. After priming, barcoded libraries were loaded onto an R9.4.1 flow cell (FLO-MIN106D) and sequenced on a MinION Mk1B device for time sufficient to achieve coverage > 100×, which allows high quality sequences. In order to ensure the highest quality of sequences, we used the Dorado v0.3.1 software with model dna_r9.4.1_e8_sup@v3.3 and set the minimum read quality (qscore) to 11 for the re-basecalling process. Demultiplexing was done using Guppy v6.5.7 software. Whole genome assembly was performed on the standalone software package Epi2me Labs with implemented wf-flu v0.0.10 workflow.

Phylogenetic analysis

For segment specific collection, duplicates of similar strains were eliminated. Sequences of known genotypes were added. Segment specific multiple alignments were generated using MAFFT (v7.450) and subsequent maximum likelihood (ML) trees were calculated with RAxML (v8.2.11) utilizing model GTR GAMMA with rapid bootstrapping and search for the best scoring ML tree supported with 1000 bootstrap replicates (**Supplementary Figure 5**). Sequences of related viruses were selected and protein-coding sequences were used for the further analysis. Time-scaled trees of were calculated with the BEAST (v1.10.4) software package using a GTR GAMMA substitution model, an uncorrelated relaxed clock with a lognormal distribution and coalescent constant population tree models. Discrete location traits were applied on country level for inferring geographical spread. Chain lengths were set to 20 million iterations and convergence checked via Tracer (v1.7.1). Time-scaled summary maximum clade credibility trees (MCC) with 10% post burn-in posterior were created using TreeAnnotator (v1.10.4) and visualized with FigTree (V1.4.4). Robustness of the MCC trees was evaluated using 95% highest posterior density (HPD) confidence intervals at each node and posterior confidence values as branch support. Spatio-temporal spread was inferred on MCC trees using SPREAD (v1.0.7) and visualized with QGIS (V3.24.3, QGIS.org)[3,4].

Cell culture

MDCK (Madin-Darby canine kidney epithelial; ATCC CCL-34 cell line), CRFK (*Felis catus*; kidney epithelial; ATCC: CCL-94) FCWF (*Felis catus*; macrophage; ATCC: CRL-2787), and Vero (*Cercopithecus aethiop*s; kidney epithelial; ATCC: CRL-1586; USA) cells were maintained in DMEM (DMEM; ThermoFisher Scientific, USA) supplemented with 5% heat-inactivated fetal bovine serum (FBS; ThermoFisher Scientific), streptomycin and penicillin (100 µg/ml and 100 U/ml, respectively). The cells were cultured in T75 flasks (TPP, Switzerland) at 37°C with 5% CO_2.

For the infection, cells were seeded in T25 flasks (TPP, Switzerland) and cultured for 24 h at 37°C with 5% CO_2. Then, cells were washed thrice with 1 x PBS and fresh DMEM medium with 2.5% trypsin (Gibco; Thermo Fisher Scientific) was applied. Cells were inoculated with 100 µl of specimen sample and cultured for 72 h at 37°C with 5% CO_2. A mock control (PBS-treated) cells were prepared in the same manner. Virus yield was assessed by titration on fully confluent Vero E6 cells in 96-well plates, according to the method of Reed and Muench[5].

Human airway epithelial cells (Epithelix SAS, Archamps, France) were maintained in BEGM medium. To develop fully differentiated human airway epithelium tissue, cells were trypsinized and seeded onto the permeable Transwell insert supports (f = 6.5 mm). The apical medium was removed after the cells reached confluence and at the same time the media was changed to ALI. Cells were cultured for 3 to 5 weeks to form well-differentiated, pseudostratified mucociliary epithelia, as previously described[6,7].

References

1. ONT Ligation sequencing influenza whole genome protocol [Internet]. [cited 2023 Jul 13]. Available from:

https://community.nanoporetech.com/docs/prepare/library_prep_protocols/ligation-sequencing-influenza-whole-genome/v/inf_9166_v109_revb_24aug2022
2. Zhou B, Donnelly ME, Scholes DT, George K St., Hatta M, Kawaoka Y, et al. Single-Reaction Genomic Amplification Accelerates Sequencing and Vaccine Production for Classical and Swine Origin Human Influenza A Viruses. J Virol [Internet]. 2009 Oct [cited 2023 Jul 13];83(19):10309. Available from: /pmc/articles/PMC2748056/
3. Mänz B, de Graaf M, Mögling R, Richard M, Bestebroer TM, Rimmelzwaan GF, et al. Multiple Natural Substitutions in Avian Influenza A Virus PB2 Facilitate Efficient Replication in Human Cells. J Virol [Internet]. 2016 Jul [cited 2023 Jul 12];90(13):5928–38. Available from: https://pubmed.ncbi.nlm.nih.gov/27076644/
4. Song W, Wang P, Mok BWY, Lau SY, Huang X, Wu WL, et al. The K526R substitution in viral protein PB2 enhances the effects of E627K on influenza virus replication. Nat Commun [Internet]. 2014 [cited 2023 Jul 12];5. Available from: https://pubmed.ncbi.nlm.nih.gov/25409547/
5. Reed LJ, Muench H. A simple method of estimating fifty per cent endpoints. Am J Epidemiol [Internet]. 1938 May 1 [cited 2023 Jul 13];27(3):493–7. Available from: https://dx.doi.org/10.1093/oxfordjournals.aje.a118408
6. Barreto-Duran E, Szczepański A, Gałuszka-Bulaga A, Surmiak M, Siedlar M, Sanak M, et al. The interplay between the airway epithelium and tissue macrophages during the SARS-CoV-2 infection. Front Immunol [Internet]. 2022 Oct 6 [cited 2023 Apr 10];13. Available from: https://pubmed.ncbi.nlm.nih.gov/36275746/
7. Milewska A, Kula-Pacurar A, Wadas J, Suder A, Szczepanski A, Dabrowska A, et al. Replication of Severe Acute Respiratory Syndrome Coronavirus 2 in Human Respiratory Epithelium. J Virol [Internet]. 2020 Jul 16 [cited 2023 Jul 13];94(15). Available from: /pmc/articles/PMC7375387/
