# Supplementary material for: Emergence and potential transmission route of avian influenza A (H5N1) virus in domestic cats in Poland, June 2023
Source: Euro Surveill. 2023 Aug 3;28(31):2300390. doi: 10.2807/1560-7917.ES.2023.28.31.2300390 (PMC10401914; doi:10.2807/1560-7917.ES.2023.28.31.2300390)
Supplement: SupplementaryFigures [file 2300390_SupplementaryFigures.pdf]

This supplementary material is hosted by *Eurosurveillance* as supporting information alongside the article "Emergence and potential transmission route of avian influenza A (H5N1) in domestic cats in Poland, June 2023", on behalf of the authors, who remain responsible for the accuracy and appropriateness of the content. The same standards for ethics, copyright, attributions and permissions as for the article apply. Supplements are not edited by *Eurosurveillance* and the journal is not responsible for the maintenance of any links or email addresses provided therein.

Supplementary figures

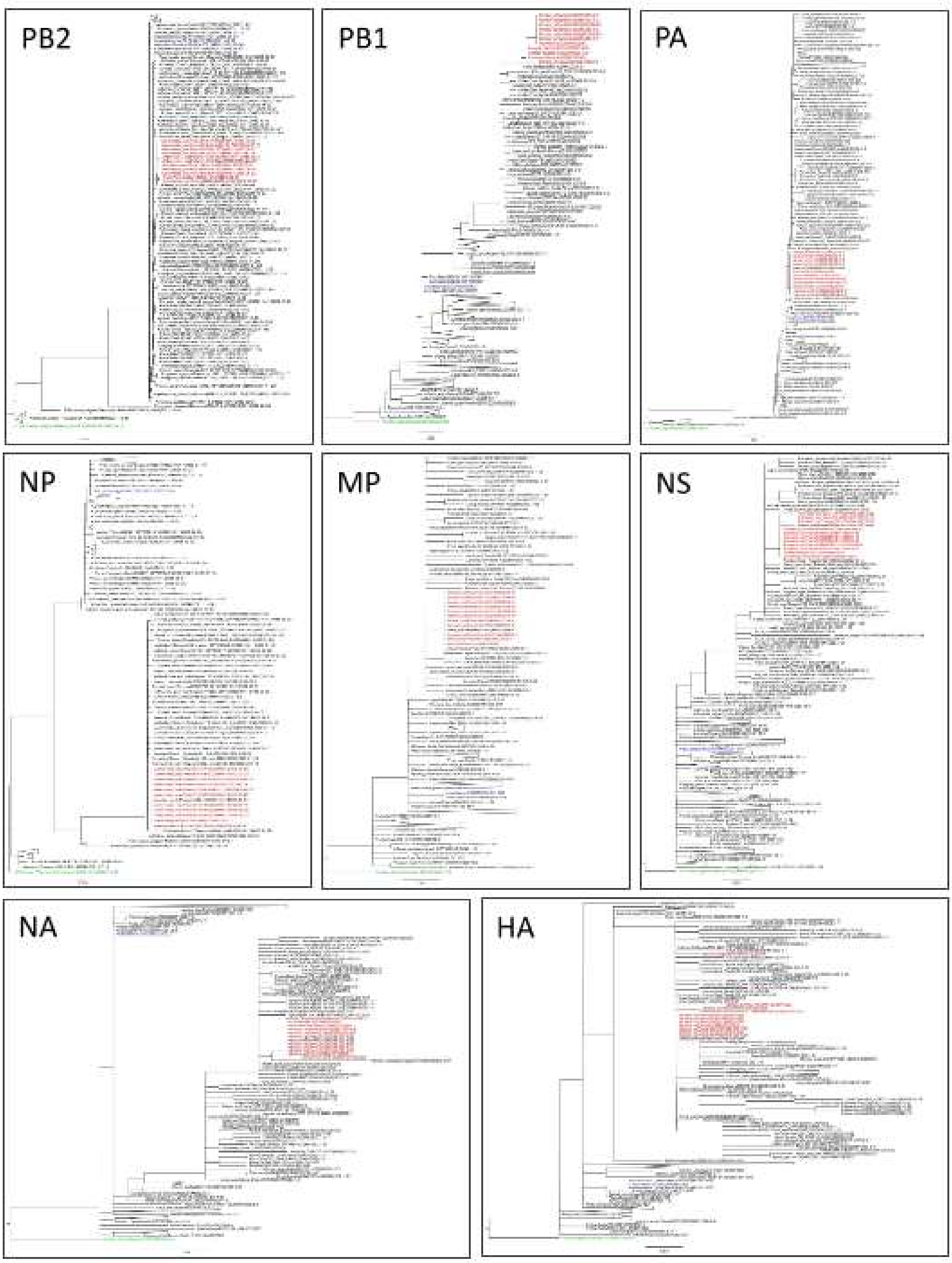

Figure S1.

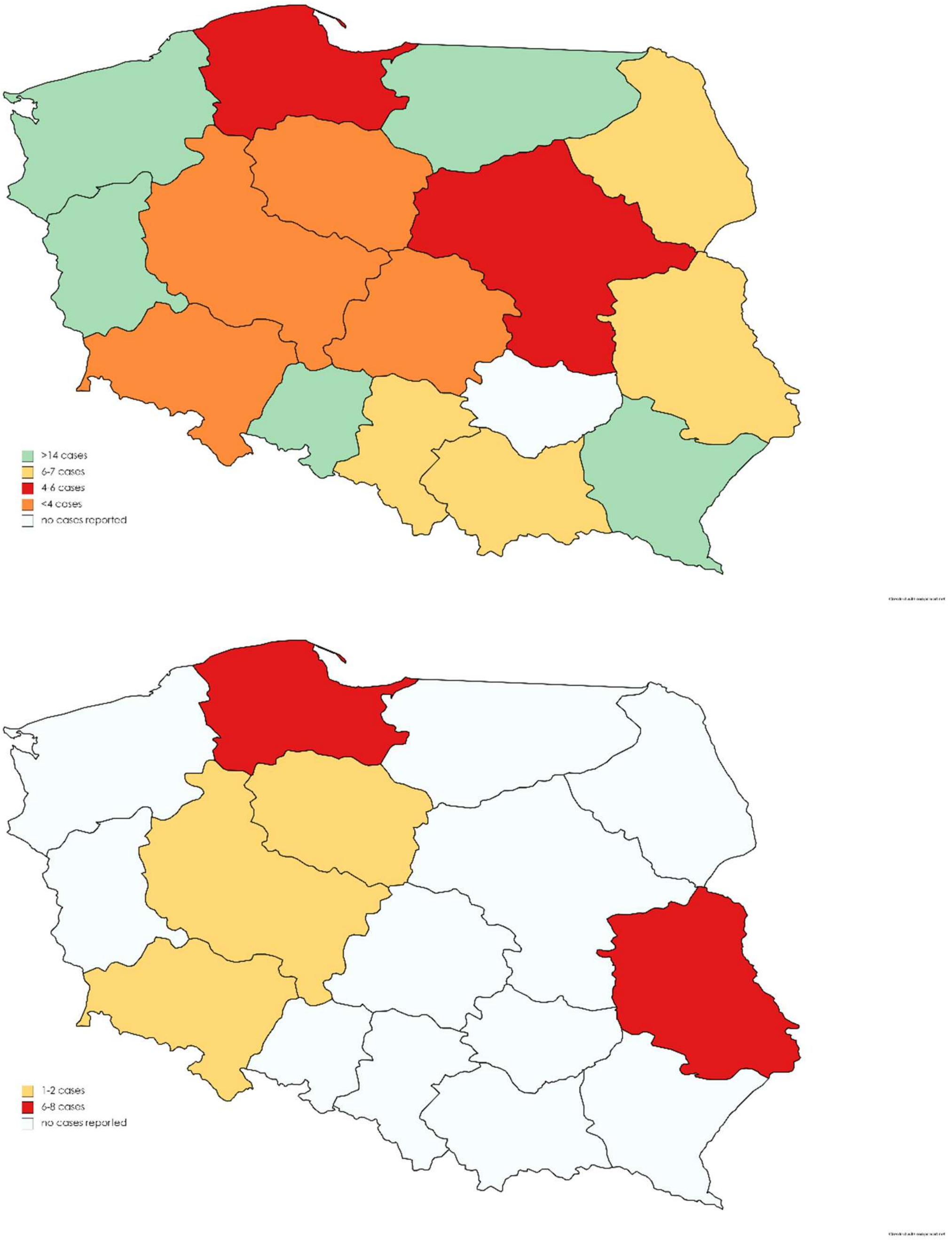
>14 cases
6-7 cases
4-6 cases
<4 cases
no cases reported
1-2 cases
6-8 cases
no cases reported

Figure S2.

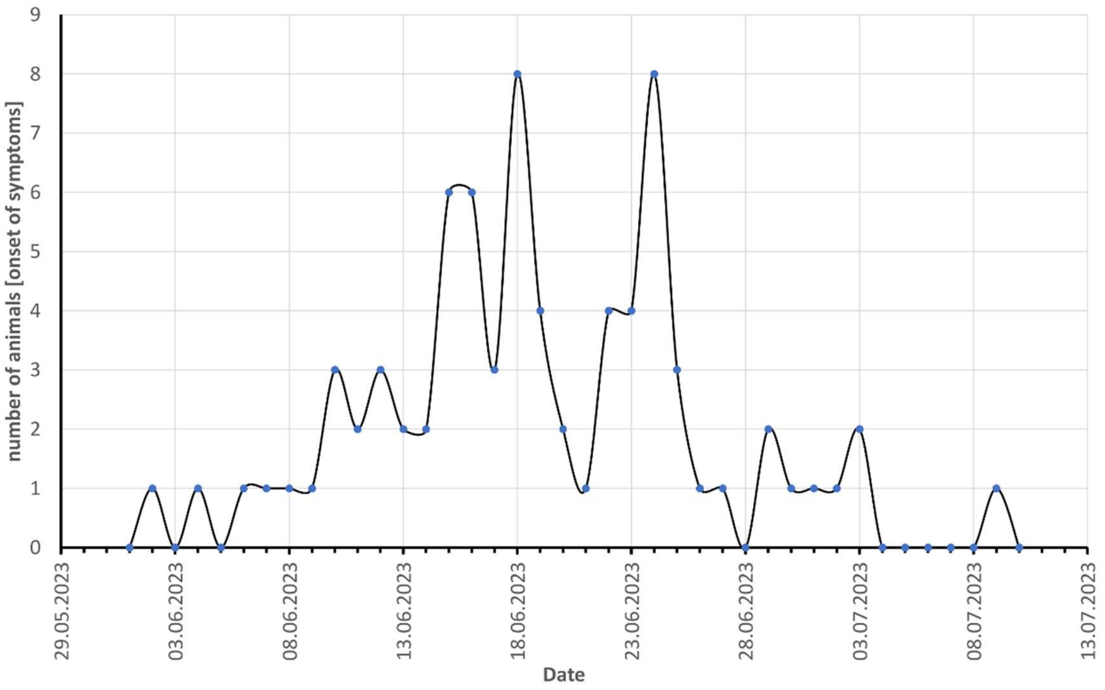

Figure S3.

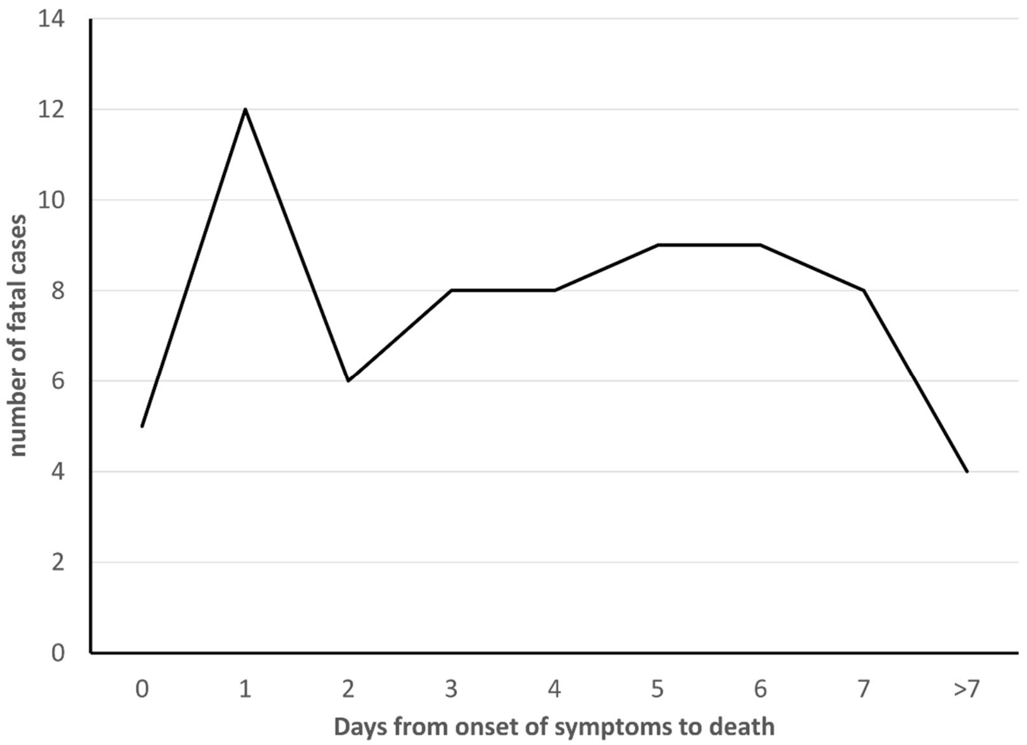

Figure S4.

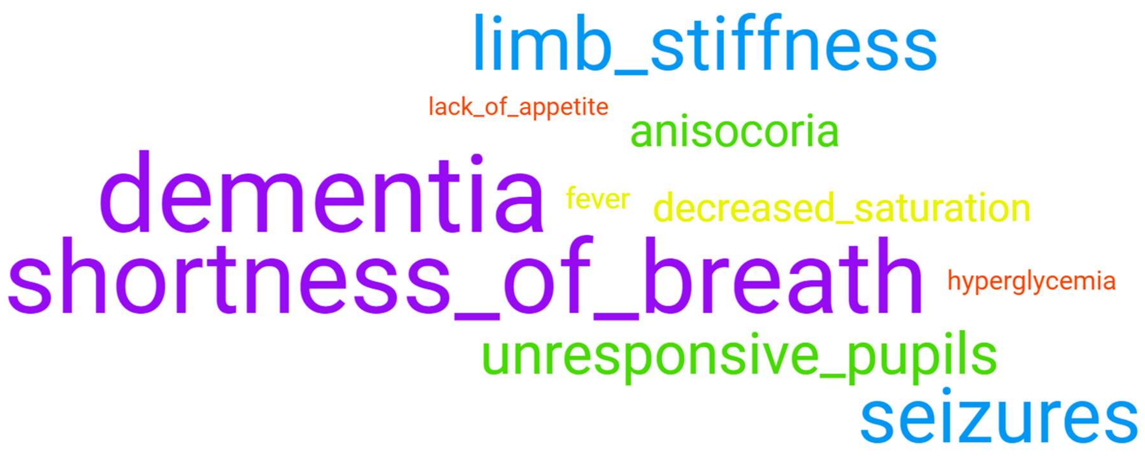
limb_stiffness
lack_of_appetite
anisocoria
dementia
fever
decreased_saturation
shortness_of_breath
hyperglycemia
unresponsive_pupils
seizures

Figure S5.
